# Exploring the Therapeutic Landscape: A Narrative Review on Topical and Oral Phosphodiesterase-4 Inhibitors in Dermatology

**DOI:** 10.3390/pharmaceutics17010091

**Published:** 2025-01-12

**Authors:** Elena Carmona-Rocha, Lluís Rusiñol, Lluís Puig

**Affiliations:** 1 Department of Dermatology, Hospital de la Santa Creu i Sant Pau, 08041 Barcelona, Spain; ecarmona@santpau.cat (E.C.-R.); lrusinol@santpau.cat (L.R.); 2 Institut de Recerca Sant Pau (IR SANT PAU), 08041 Barcelona, Spain; 3 Unitat Docent Hospital Universitari Sant Pau, Facultat de Medicina, Universitat Autònoma de Barcelona, 08041 Barcelona, Spain

**Keywords:** phosphodiesterase-4, PDE4, apremilast, roflumilast, orismilast, mufemilast, difamilast, psoriasis, atopic dermatitis, topical therapy, oral therapy, off-label

## Abstract

Phosphodiesterase-4 (PDE4) is involved in the synthesis of inflammatory cytokines that mediate several chronic inflammatory disorders, including psoriasis and atopic dermatitis. In recent years, the therapeutic armamentarium in dermatology has expanded with the introduction of PDE4 inhibitors, both in oral and topical formulations. PDE4 inhibitors have gained increasing interest due to their remarkable safety record and ease of prescription, as evidenced by the recent influx of literature detailing its off-label uses. Apremilast was the first PDE4 inhibitor approved by the US Food and Drug Administration (FDA) and the European Medicines Agency (EMA) for psoriasis, psoriatic arthritis, and oral ulcers of Behcet’s disease. Off-label use has been reported in diverse dermatological conditions, including aphthous stomatitis, chronic actinic dermatitis, atopic dermatitis, cutaneous sarcoidosis, hidradenitis suppurativa, lichen planus, and discoid lupus erythematosus. Roflumilast is a PDE4 inhibitor that was approved by the FDA and the EMA as an oral treatment of chronic obstructive pulmonary disease. Since patent expiration, several generic formulations of oral roflumilast have become available, and various studies have documented its off-label use in psoriasis and other dermatological conditions such as hidradenitis suppurativa, recurrent oral aphthosis, nummular eczema, lichen planus, and Behçet’s disease. Topical roflumilast has received FDA approval for treatment of plaque psoriasis and seborrheic dermatitis. The favorable safety profile encourages its long-term use as an alternative to corticosteroids, addressing the chronic nature of many dermatological conditions. New oral PDE4 inhibitors are being developed, such as orismilast (LEO-32731), mufemilast (Hemay005), difamilast (OPA-15406) or lotamilast (E6005/RVT-501), among others. This narrative review provides a comprehensive synthesis of the pharmacology, clinical efficacy, safety profile, and practical considerations regarding the oral and topical use of PDE4 inhibitors in dermatology.

## 1. Introduction

Chronic immune-mediated inflammatory skin conditions, such as atopic dermatitis (AD), psoriasis, and hidradenitis suppurativa, are estimated to collectively affect approximately 20–25% of the global population [1]. In addition to the challenges of managing a lifelong chronic skin condition, these patients frequently face an elevated risk of comorbidities, including cardiovascular disease, and increased mortality [2]. The advent of biologic therapies and small molecule drugs has transformed the treatment landscape for chronic inflammatory skin diseases. However, their high cost represents a significant limitation, particularly when used off-label, and some patients tend to prefer safe and effective oral medications [3,4,5].

Several oral immunosuppressive drugs, including methotrexate, cyclosporine, Janus kinase (JAK) inhibitors, and an allosteric TYK2 inhibitor, are available for treatment of psoriasis and AD. However, these treatments require careful monitoring for potential risks such as hepatotoxicity (methotrexate), nephrotoxicity (cyclosporine), and serious adverse events, including infections, thrombosis, cancer, and major cardiovascular complications that can develop in selected populations treated with JAK inhibitors [6,7,8].

Phosphodiesterase-4 (PDE4) inhibitors have been established as effective therapeutic options for treating inflammatory conditions, both in oral and topical formulations. Apremilast, roflumilast and crisaborole have been sequentially approved for the treatment of inflammatory skin diseases. Apremilast was the first PDE4 inhibitor and is currently US Food and Drug Administration (FDA) and European Medicines Agency (EMA)-approved for psoriasis, psoriatic arthritis, and oral ulcers of Behcet’s disease [9]. Roflumilast is a PDE4 inhibitor that was approved by the FDA and the European Medicines Agency (EMA) as an oral treatment of chronic obstructive pulmonary disease [10]. In dermatology, topical roflumilast has received FDA authorization for treatment of plaque psoriasis, seborrheic dermatitis and AD [11,12]. Crisaborole is a topical PDE4 inhibitor approved by the FDA and EMA for the treatment of AD [13]. PDE4 inhibitors have garnered increasing interest due to their remarkable safety record and ease of prescription, as demonstrated by the growing body of literature highlighting their off-label applications, including aphthous stomatitis, psoriasis, AD, hidradenitis suppurativa, sarcoidosis, lichen planus, discoid lupus erythematosus, and others [14,15].

New oral PDE4 inhibitors are being developed, such as orismilast (LEO-32731), mufemilast (Hemay005), difamilast (OPA-15406) or lotamilast (E6005/RVT-501), among others. This narrative review provides a comprehensive synthesis of the pharmacology, clinical efficacy, safety profile, and practical considerations regarding the use of topical and oral PDE4 inhibitors in dermatology.

## 2. Methods

For this narrative review, we performed an electronic search of English-language medical literature on PubMed, covering the period from January, 2014, to October, 2024. The research involved matching the following terms: “phosphodiesterase-4”, “inhibitors”, “apremilast”, “CC-10004”, “roflumilast”, “crisaborole”, “AN2728”, “orismilast”, “IBI353”, “mufemilast”, “Hemay005”, “difamilast”, “OPA-15406”, “ME3183”, “lotamilast”, “E6005”, “RVT-501”, “LEO-29102”, “pefcalcitol”, “M5181”, “zatolmilast”, “PF-07038124”, “HFP034”, “GW842470X”, “AWD-12-281”, “DRM02”, “oral”, “topical”, “dermatology”. Similarly, a search using the same terms was conducted on clinicaltrials.gov. The articles were screened based on their abstracts and selected for relevance after reviewing the studies. We included only human studies, such as clinical trials, reviews, systematic reviews and case series. Additionally, we conducted online Google searches to identify international nonproprietary names and investigational drug names.

## 3. Mechanism of Action of PDE4 Inhibitors

Phosphodiesterases (PDEs) are key enzymes that regulate intracellular cyclic nucleotide metabolism by breaking down cyclic adenosine monophosphate (cAMP) or cyclic guanosine monophosphate (cGMP) [16]. At present, eleven different families of isoenzymes of PDE exist in mammals that show a distinct species-specific and organ-specific distribution. For example, PDE3 is preferentially expressed in the heart, lungs, liver, platelets, adipose tissue, and inflammatory cells [17]. Regarding PDE4, four subtypes exist (PDE4A, PDE4B, PDE4C, and PDE4D), leading to up to 20 variants of PDE4 due to genetic splicing [18]. PDE4,a cAMP-specific hydrolase, is expressed in immune cells such as basophils, mast cells, eosinophils, B and T lymphocytes, monocytes, macrophages, neutrophils, as well as endothelial cells [19]. Additionally, dermal fibroblasts of patients with AD exhibit enhanced expression of all PDE4 (A-D) variants [20,21,22].

Cyclic AMP is a crucial second messenger involved in controlling various cellular processes, including cell trafficking, the release of inflammatory mediators, and immune cell proliferation. When cAMP levels decrease, immune functions of T cells, monocytes, macrophages, and neutrophils become activated [23,24].

**Figure 1:**
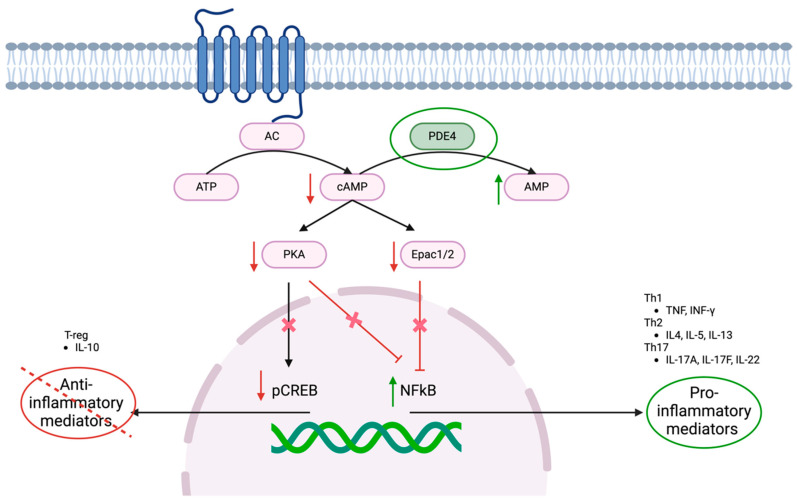
Pro-inflammatory state: Lower levels of cAMP prevent the activation of PKA and Epac1/2, leading to increased NF-κB activity and the induction of pro-inflammatory mediators. Additionally, the activation of pCREB by PKA is reduced, leading to a decrease in the expression of anti-inflammatory mediators. Abbreviations: AC, Adenylate cyclase; PDE4, phosphodiesterase 4; PKA, protein kinase A; Epac1/2, exchange protein 1/2 activated by cAMP; pCREB, phosphorylated cAMP-responsive element binding protein; NF-kB, nuclear factor kappa-light chain-enhancer of activated B cells. Created with www.biorender.com (accessed on 9 January 2025).

Intracellular levels of cAMP are mainly regulated by adenyl cyclase (AC) and PDE4. AC catalyzes the conversion of adenosine triphosphate (ATP) into cAMP and pyrophosphate, whereas PDE4 hydrolyzes the 3′,5′ phosphodiester bond of cAMP to yield 5′adenosine monophosphate (5′-AMP) and consequently reduces intracellular cAMP levels. Low levels of cAMP prevent the activation of protein kinase A (PKA) and exchange proteins 1 and 2 activated by cAMP (Epac1/2), which, in turn, allows the activation of nuclear factor kappa B (NF-κB), promoting the production of pro-inflammatory mediators. Furthermore, the suppression of PKA-induced phosphorylation of cAMP-responsive element-binding protein (pCREB) leads to a reduction in the transcription of anti-inflammatory mediators. (Figure 1) [18,23,24].

**Figure 2:**
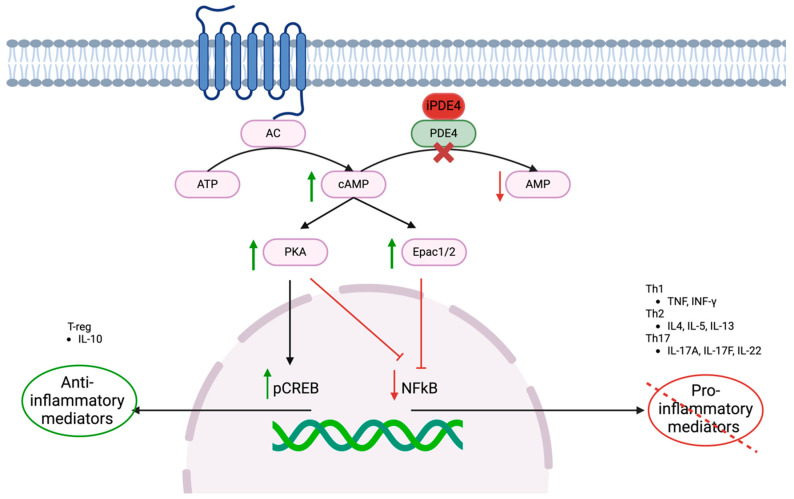
PDE4 inhibitors’ mechanism of action: Upon inhibition of PDE4, intracellular levels of cAMP rise, leading to the activation of PKA and Epac1/2 and the subsequent inhibition of NF-kB. In addition, pCREB levels increase, promoting the production of anti-inflammatory mediators. Abbreviations: AC, Adenylate cyclase; PDE4, phosphodiesterase 4; PKA, protein kinase A; Epac1/2, exchange protein 1/2 activated by cAMP; pCREB, phosphorylated cAMP-responsive element binding protein; NF-kB, nuclear factor kappa-light chain-enhancer of activated B cells; iPDE4, inhibitor of PDE4. Created with www.biorender.com (accessed on 9 January 2025).

Thus, by preventing the degradation of cAMP, PDE4 inhibitors enable PKA and Epac1/2 to suppress NF-κB-related inflammatory mediators while promoting anti-inflammatory pathways, such as PKA-mediated pCREB activation (Figure 2) [24].

Adenylate cyclase is upregulated in mononuclear leukocytes in AD, leading to increased cAMP production [25]. This rise in cAMP is thought to drive heightened PDE activity as part of an effort to maintain homeostasis in unstimulated cells. When these leukocytes are stimulated, the expected further increase in cAMP levels would typically dampen the cell response. However, due to the already elevated PDE activity, maintaining sufficient cAMP levels becomes difficult [25]. Additionally, increased PDE activity in atopic monocytes has been linked to higher prostaglandin E2 production, which in turn raises levels of IL-6 and IL-10, both of which contribute to the imbalance between Th1 and Th2 cells [26]. Although IL-10 is generally regarded as a suppressor of immune responses, its elevated levels in AD monocytes are associated with reduced IFNγ production by Th1 cells, shifting the Th1/Th2 balance toward a Th2-dominant response [26].

PD4 inhibitors have been shown to suppress the production of several pro-inflammatory cytokines, such as tumor necrosis factor (TNF) in T cells, natural killer (NK) cells and dendritic cells; interleukin (IL)-2 and IL-5 in T cells, IL-12 in monocytes, interferon-γ (IFN)γ in T cells and NK cells, and IL-8, leukotriene B4 (LTB4) and the adhesion molecule CD18/CD11b (Mac-1) in neutrophils; furthermore, the expression of the anti-inflammatory cytokine IL-10 becomes augmented in monocytes [19,27].

Studies in psoriatic skin have shown that the overexpression of PDE4 promotes the production of proinflammatory cytokines and the proliferation of immune cells. In contrast, inhibiting PDE4 increases cAMP levels, leading to a reduction in the expression of key cytokines involved in the pathophysiology of psoriasis, including TNF, IFN-γ, IL-17, and IL-23 [28]. In a phase 3 placebo-controlled study, patients with moderate to severe psoriasis were treated with apremilast. At week 16, psoriasis area severity index (PASI) value improvement correlated with the reduction of plasmatic levels of IL-17A, IL-17F, and IL-22 [29]. Additionally, in a pharmacodynamics study, upon treatment of psoriatic arthritis patients with apremilast, plasma levels of IL-6, IL-8, and TNF were reduced after 24 weeks of treatment, and those of IL-17 and IL-23 levels by 40 weeks [30].

## 4. Oral and Topical Approved Inhibitors of PDE4

### 4.1. Apremilast (CC-10004; Otezla^®^)

Apremilast is an orally administered small molecule that specifically inhibits PDE4, modulating the immune system by increasing intracellular levels of cAMP. It is approved by the Food and Drug Administration (FDA) and the European Medicines Agency (EMA) for the treatment of psoriasis, psoriatic arthritis, and oral ulcers in Behçet’s disease (Table 1) [14]. However, due to its efficacy, safety profile, and tolerability, it has been tested in several other diseases, such as hidradenitis suppurativa, lichen planus, and AD, among others. The most common side effects reported are nausea, diarrhea, headache, upper respiratory tract infections, and nasopharyngitis [14].

Its oral bioavailability is 73%, and it is metabolized catabolically via multiple pathways, including the cytochrome P450 (CYP) pathway [48]. Only 3% of the dose is excreted unchanged in urine. Its bioavailability is reduced when administered concomitantly with CYP inducers, such as rifampicin. In fact, Liu et al. demonstrated that apremilast could be administered alongside methotrexate without affecting the pharmacokinetic exposure to either drug [49].

#### 4.1.1. Apremilast in Psoriasis

Apremilast showed promising results in clinical trials and became the first PDE4 inhibitor approved for the treatment of a cutaneous disease, namely psoriasis in 2014 [33,34]. Moreover, in April and October 2024, apremilast was approved by the FDA and EMA, respectively, for the treatment of moderate to severe psoriasis in pediatric patients aged 6 or older and weighing ≥ 20 kg [50].

The ESTEEM trials, two double-blind phase 3 trials evaluated apremilast efficacy and safety in patients affected of moderate to severe psoriasis [33,34,51]. A total of 844 and 411 patients were included in the ESTEEEM 1 and ESTEEM 2 trials, respectively. Patients were randomized to receive apremilast 30 mg twice daily or a placebo for 16 weeks. At week 16, those initially assigned to the placebo group switched to apremilast. At week 32, patients originally randomized to apremilast who had achieved at least a PASI75 [33] or 50% (PASI50) [34] reduction in their baseline PASI score were re-randomized to continue apremilast or switch to placebo. For patients re-randomized to placebo at week 32, apremilast treatment was resumed upon losing their PASI75 response in the ESTEEM 1 trial or upon losing 50% of their PASI improvement from week 32 in the ESTEEM 2 trial. Blinding was maintained through week 52, after which patients could opt to continue apremilast treatment for up to four additional years in a long-term extension phase.

In the ESTEEM 1 trial, 61% of the 77 patients re-randomized to apremilast at week 32 achieved a PASI75 response by week 52. Among the 77 patients re-randomized to placebo at week 32, 13 (17%) maintained their PASI75 response without resuming apremilast before week 52. The remaining 64 patients lost their PASI75 response and restarted apremilast, with 70% regaining their PASI75 response before week 52 [33].

In the ESTEEM 2 trial, 80% of the 61 patients re-randomized to apremilast at week 32 achieved a PASI50 response by week 52. Among the 62 patients re-randomized to placebo at week 32, 30 (48%) retained more than 50% of their PASI improvement from week 32 and did not require apremilast retreatment before week 52. Of the 32 patients who lost 50% or more of their PASI improvement while on placebo, 21 (66%) regained a PASI50 response after resuming apremilast before week 52 [34].

Apremilast was also evaluated for the management of psoriatic arthritis in the PALACE 1 [52], PALACE 2 [31], PALACE 3 [32], and PALACE 4 [53] trials. Patients included in PALACE 4 were naïve to disease-modifying antirheumatic drug (DMARD), contrary the remaining PALACE trials included DMARD-experienced patients. Patients were randomized to receive apremilast 20 mg or 30 mg twice daily (BID) or placebo BID for 24 weeks. At week 24, patients in the placebo group were assigned to apremilast 20 mg or 30 mg BID. Blinded treatment continued until week 52. Apremilast demonstrated efficacy in improving signs and symptoms in both DMARD-experienced and DMARD-naïve patients with active psoriatic arthritis. At week 16, the ACR20 response rate was significantly higher with apremilast 30 mg twice daily compared to placebo, in both DMARD-experienced patients (32–41% vs. 18–19%) [31,32,52] and DMARD-naïve patients (31% vs. 16%) [53].

Apremilast consistently demonstrated good tolerability across all clinical trials involving patients with psoriasis and psoriatic arthritis [51]. Crowley et al. reported safety results after more than 156 weeks of follow-up for the 1184 patients enrolled in the ESTEEM 1 and ESTEEM 2 trials [54]. The most frequently reported adverse events (AE)s were diarrhea, nausea, upper respiratory tract infections, nasopharyngitis, tension headache, and headache.

Ten years after its approval, several studies have evaluated the real-world results of apremilast. Table 2 highlights results of the most significant real-world studies [55,56,57,58]. A Spanish retrospective study included 377 patients: 292 with moderate to severe psoriasis and 85 with palmoplantar psoriasis [55]. Drug survival rate after 1 year of treatment was 54.9%. PASI75 and PASI90 responses were achieved in 49.7% and 26.5% of patients, respectively [55]. Better results were reported in an Italian real-world study involving 231 patients treated with apremilast 30 mg BID [58]. After 52 weeks of treatment, PASI75, PASI90, and PASI100 were achieved in 97%, 89.3%, and 62% of patients, respectively.

Regarding apremilast’s safety and tolerability, it has always been a notable concern. E. de Alcázar et al. reported a 45.1% discontinuation rate after one year of treatment [55]. Nearly one-quarter (23.9%) of patients discontinued due to loss of efficacy, while 15.9% stopped treatment because of AEs, making this the second major cause for discontinuation. The most commonly reported adverse events include gastrointestinal issues such as nausea and diarrhea, weight loss, and headache [55]. Furthermore, cases of worsening depression, suicide attempts, suicidal ideation, and suicidal behavior had been reported in clinical trials and post-marketing surveillance. To address this concern, P. Vakharia et al. analyzed data from real-world adverse event reporting systems and a large, urban, single-center U.S. patient population. Their findings revealed no safety signal linking apremilast to suicidality [59].

A phase 4 clinical trial (NCT03082729) aimed to evaluate the impact of apremilast treatment on aortic vascular inflammation, cardiometabolic biomarkers, and body composition in patients with moderate to severe psoriasis. This effect was assessed using fluorodeoxyglucose (FDG) positron emission tomography (PET) combined with computed tomography (CT). A total of 70 patients received treatment over a 16-week period. At the end of this period, vascular inflammation in the aorta was compared to baseline levels. The level of aortic inflammation was assessed using the tissue-to-background ratio (TBR) of the standardized uptake value (SUV) and was then compared to baseline SUV values. Patients showed a reduction in SUV of 0.017 (*p* = 0.612), which was not statistically significant. Additionally, comparisons of ferritin levels at baseline and 16 weeks showed a non-significant decrease, as did comparisons of C-reactive protein (CRP) levels.

Apremilast has also been evaluated for the treatment of palmoplantar pustulosis in two clinical trials (NCT04572997, NCT04057937).

The APLANTUS trial (NCT04572997) was a multicenter, single-arm, phase 2 study that included 21 patients with moderate to severe palmoplantar pustulosis, who were treated with apremilast 30 mg BID for 20 weeks. At the end of the treatment period, the baseline and 20-week palmoplantar pustulosis area and severity index (PPPASI) scores were compared. Patients showed a reduction in PPPASI from 16.5 to 7.65. Additionally, 61.9% of patients achieved PPPASI50, and 14.2% achieved PPPASI75. Only one patient discontinued treatment due to AEs. The most common AEs were headache, nausea, and diarrhea, with no serious AEs reported.

The NCT04057937 trial was a multicenter, randomized, double-blind, placebo-controlled, phase 2 study. A total of 90 patients with moderate to severe palmoplantar pustulosis were treated with either apremilast or placebo for 16 weeks, followed by 16 weeks of apremilast for all participants. After the initial 16-week period, PPPASI50 was achieved by 40.9% of the placebo group and 78.3% of the apremilast group. PPPASI75 was achieved by 15.9% of participants in the placebo group and 43.5% of those in the apremilast group. Most AEs were mild, with nausea and diarrhea being the most reported.

#### 4.1.2. Apremilast in Behçet’s Disease

Apremilast 30 mg BID was evaluated for the treatment of oral ulcers in patients affected by Behçet’s disease. Compared to placebo, patients treated with apremilast showed significantly fewer oral ulcers (primary endpoint) and a significant reduction in oral ulcer pain after 12 weeks of treatment in phase 2 and phase 3 trials [60,61]. Apremilast also exhibited significantly faster resolution and healing time (2.1 weeks vs. 8.1 weeks) compared to placebo [61]. Apremilast was approved in 2019 by the FDA for the treatment of oral ulcers associated with Behçet’s disease [36].

Apremilast has also exhibited significant efficacy for the treatment of oral ulcers in Behçet’s disease in real-world settings. Patients with oral and/or genital ulcers refractory or intolerant to conventional therapies and biologics showed a strong response to apremilast 30 mg BID. Atienza-Mateo et al. reported outcomes in 47 patients, with up to 89% presenting complete or partial resolution of ulcers after 1–2 weeks of treatment [62]. Additionally, patients followed for 3 months (n = 37), 12 months (n = 12), and 24 months (n = 2) achieved complete or partial responses in 89%, 100%, and 100% of cases, respectively [62].

Finally, apremilast was compared to TNF inhibitors in a retrospective analysis [63]. A total of 78 patients were included and equally located to apremilast or TNF inhibitors treatments. Both groups experienced significant reductions in oral ulcers at 3 months (*p* < 0.001) and 6 months (*p* = 0.01), with no significant differences observed between the treatments. Both treatments were equally effective in reducing genital ulcers, while TNF inhibitors showed superior efficacy in managing articular involvement. Moreover, apremilast had a higher discontinuation rate due to gastrointestinal side effects [63].

Currently, there are several clinical trials evaluating apremilast for psoriasis and for vascular inflammation associated with psoriasis (NCT03082729).

#### 4.1.3. Off-Label Uses of Apremilast

Because of its oral availability and safety profile, apremilast has been tested in a wide spectrum of diseases, such as AD, hidradenitis suppurativa, lichen planus, and cutaneous lupus erythematosus, among others.

##### Off-Label Apremilast in Atopic Dermatitis

A phase 2 placebo-controlled trial evaluated the efficacy, safety, and pharmacodynamics of apremilast in adults with moderate to severe AD [37]. A total of 185 patients were randomly allocated to receive either apremilast 30 mg BID, apremilast 40 mg BID, or a placebo for 12 weeks. From weeks 12 to 24, all patients received either apremilast 30 mg or 40 mg BID. At week 12, the apremilast 40 mg twice-daily group showed a statistically significant improvement in the Eczema Area and Severity Index (EASI) compared to the placebo group, with a 31.6% vs. 11% improvement in EASI percent change from baseline, respectively. However, the EASI improvement experienced by patients in the apremilast 30 mg twice-daily group was not significantly different from placebo. EASI-50 response was achieved by 32.8%, 31%, and 42.9% of the placebo, apremilast 30 mg, and apremilast 40 mg groups, respectively. None of the apremilast dosages exhibited statistically significant differences compared to placebo in terms of EASI-50 achievement. AEs with apremilast 30 mg BID were consistent with the known profile of the drug, including nausea, diarrhea, headache, and nasopharyngitis. However, patients treated with apremilast 40 mg BID experienced a higher frequency of AEs, including cellulitis. The apremilast 40 mg dosage was discontinued during the trial due to these AEs [37].

Cunningham et al. evaluated the efficacy of apremilast when added to dupilumab in a phase 2, prospective, open-label trial [64]. A total of 10 patients were included and treated with dupilumab 300 mg combined with apremilast 30 mg BID for 16 weeks. The mean percent change from baseline in BSA and EASI was −37.6 ± 26.6% and −32.6 ± 42.6%, respectively. Eight patients experienced mild AEs, the most common being nausea, diarrhea, and headache [64].

##### Off-Label Apremilast in Hidradenitis Suppurativa

In a randomized placebo-controlled trial, 20 patients with moderate hidradenitis suppurativa (HS) were assigned to receive apremilast 30 mg BID or placebo in a 3:1 ratio for 16 weeks [38]. The primary outcome was the Hidradenitis Suppurativa Clinical Response (HiSCR) at week 16, which was achieved by 0% and 53.3% of the placebo and apremilast groups, respectively. Patients treated with apremilast (n = 15) experienced a significant reduction in total abscess and nodule (AN) count, along with decreased pain and itching compared to the placebo group. Mild to moderate headaches and gastrointestinal symptoms were reported as side effects in the apremilast group, but no patients discontinued the treatment [38].

Of the 8 patients who achieved HiSCR, only 4 remained on treatment after two years. Two patients discontinued due to active pregnancies, one discontinued because of intolerable nausea, and the remaining patient stopped apremilast on her own after experiencing no further HS symptoms. The four patients who remained on apremilast maintained their HiSCR score at both 1 and 2 years of follow-up [65].

##### Other Off-Label Uses of Apremilast

Apremilast has also been tried in patients with lichen planus [39]. A single center, non-randomized, open-label trial included 34 patients, of which 26 completed the study. After 12 weeks, the Physician Global Assessment (PGA) of disease improved 2 or more grades in 34.61% (9/26) of patients to. Additionally, 42.30% (11/26) achieved more than 50% improvement in their lesions based on the Subject Global Assessment. Only 6 patients experienced one or more AEs related to apremilast, headache being the most common [39].

In 2008, a phase 2, open-label, single-arm study evaluated apremilast in 8 patients with discoid lupus erythematosus [40]. Patients were treated with apremilast 20 mg BID for 85 days and showed a significant (*p* < 0.05) reduction in the Cutaneous Lupus Erythematosus Disease Area and Severity Index (CLASI) [40].

Apremilast 20 mg BID was evaluated in chronic cutaneous sarcoidosis with modest results [42]. Additionally, Koschitzky et al. reported 5 patients with morphea who were successfully treated with apremilast [41]. Single case reports of apremilast use are numerous, such as a successfully treated patient with psoriasis and anti-laminin γ1 (p200) pemphigoid [43]. Other diseases in which apremilast has been tested are central centrifugal cicatricial alopecia, yielding modest results (NCT03521687); vulvodynia, where no improvement was observed in patients (NCT00814632), dermatomyositis (NCT03529955), erythema nodosum leprosum [44], pemphigus foliaceous and vulgaris [45,46], and Hailey-Hailey [47], among others. Currently, several ongoing clinical trials are evaluating apremilast in a wide spectrum of inflammatory diseases (Table 3).

### 4.2. Roflumilast

#### 4.2.1. Oral Roflumilast

Roflumilast (Daxas^®^, Daliresp^®^) is a selective PDE4 inhibitor. It was initially approved in 2011 by the EMA and the FDA to reduce the risk of exacerbations in chronic obstructive pulmonary disease (COPD) and chronic bronchitis phenotype [10]. According to IC50 values reflecting inhibitor affinity for the PDE4 enzyme, roflumilast exhibits 25 to 300 times greater potency than either apremilast or crisaborole, depending on the comparator and the specific PDE4 isoform analyzed [66].

Oral roflumilast is fully absorbed in the gastrointestinal tract and has a bioavailability of approximately 80% [67]. Roflumilast is primarily processed in the liver by the cytochromes CYP1A2 and CYP3A4, which convert the compound into the active metabolite roflumilast N-oxide [68]. This metabolite accounts for over 90% of roflumilast’s total PDE4 inhibitory activity [68].

No dosage adjustment is required for elderly patients or those with renal impairment. However, roflumilast is contraindicated in patients with moderate to severe liver impairment (Child-Pugh B or C) and should be used with caution in cases of mild liver impairment [15].

Overall, roflumilast is well tolerated and demonstrates an acceptable safety profile. The most frequently observed AEs are those typically associated with PDE4 inhibitors, primarily gastrointestinal disturbances and weight loss [69]. A pooled analysis of more than 6000 COPD patients treated with roflumilast in clinical trials found that the overall incidence of AEs was comparable to that observed in the placebo group, with higher incidences of diarrhea, weight loss, nausea, headache, back pain, insomnia, reduced appetite, and dizziness compared to the placebo group [69].

In clinical trials for COPD and chronic bronchitis, the most common AEs were diarrhea (8–9%), weight loss (6–12%), and nausea (5%) [70]. Nasopharyngitis was reported in 5–8% of cases, and 4% experienced upper respiratory tract infections, though these rates were similar to those in the placebo group [70]. Most AEs occurred during the first weeks of treatment and were self-limiting. Approximately 15% of patients discontinued treatment due to side effects (compared to 9% in the placebo group), with diarrhea and nausea being the most frequent reasons for discontinuation [70]. Regarding weight loss, its incidence was nearly double in the roflumilast group (67.4% vs. 37.7% in the placebo group). Significant weight loss, defined as more than 10% of baseline weight, was observed in 7.1% of the treatment group compared to 1.9% in the control group [70]. This weight loss has been associated with an improvement in the glycemic metabolic profile and represents a potential therapeutic tool for obesity, insulin resistance, and metabolic syndrome [71].

Early reports also suggested increased rates of acute pancreatitis, psychiatric symptoms (including anxiety, depression, insomnia and suicide), and even prostate, lung, and colorectal cancers [70,72]. However, these initial findings could not be confirmed, and later studies have shown that these risks are minimal. In fact, roflumilast may even hold promise as a potential treatment for cognitive decline, Alzheimer’s disease, or schizophrenia [15].

##### Off-Label Oral Roflumilast in Psoriasis

Until 2023, the efficacy of oral roflumilast in treating psoriasis had been minimally documented in the literature, with reports largely limited to clinical case studies [73,74,75,76,77,78].

The efficacy and safety of oral roflumilast for psoriasis has been recently studied in a company-independent, multicenter, randomized, double-blind, placebo-controlled phase 2 trial (PSORRO trial; NCT04549870) conducted in 2023 [79]. A total of 46 patients were randomized 1:1 to receive either oral roflumilast 500 μg once daily (QD) or placebo. The primary endpoint of PASI75 was met, with 35% of participants achieving this level of improvement compared to 0% in the placebo group at week 12. At week 12, patients in the placebo group were transitioned to open-label roflumilast until week 24. The safety and efficacy profile was maintained over a 24-week period. The most common AEs included gastrointestinal symptoms, weight loss, headaches, and insomnia, resulting in therapy discontinuation for three patients (roflumilast n = 2; placebo, n = 1).

Post-hoc analyses from the PSORRO study evaluated the effects of oral roflumilast on body weight and cardio-metabolic parameters in patients with psoriasis [80]. Body weight, blood pressure, gastrointestinal symptoms, and laboratory tests were assessed without any lifestyle or dietary changes. The baseline characteristics of the groups were similar, with a mean weight of 103.6 kg. Patients on roflumilast experienced a median weight loss of −2.6% at week 12 and −4% at week 24, compared to negligible changes in the placebo group. Reduced appetite was more common with the treatment, but no significant alterations in blood pressure or lab tests were observed.

Patient-reported outcomes obtained from the PSORRO trial have also been reported [81], showing high levels of treatment satisfaction, with significant improvements in DLQI and and numeric rating scale for itch and skin pain over 24 weeks.

A recent randomized, controlled study compared oral roflumilast with intramuscular methotrexate (MTX) for patients with plaque and scalp psoriasis (NCT05684744) [82]. In this study, 30 adult patients were randomly assigned in a 1:1 ratio to receive either oral roflumilast 500 μg or weekly intramuscular MTX at 0.2–0.4 mg/kg. The primary endpoints of the study included the percentage of patients achieving PASI 75 and scalp-modified PASI (SmPASI). After 12 weeks, both groups demonstrated a statistically significant reduction in all assessed scores. A higher percentage of patients in the roflumilast group achieved PASI 75 (67% vs. 60%), PASI 90 (47% vs. 27%), PASI 100 (27% vs. 7%), SmPASI 75 (87% vs. 57%), SmPASI 90 (60% vs. 50%), and SmPASI 100 (53% vs. 36%), although the differences did not reach statistical significance. AEs were reported in 60% and 46.7% of patients receiving roflumilast and MTX, respectively. In the roflumilast group, most symptoms were gastrointestinal, while the MTX group primarily experienced gastric upset, fatigue, and anemia. The AEs were manageable and did not require stopping treatment for either group.

These findings suggest that oral roflumilast may hold significant promise as a new treatment option for psoriasis. With the availability of generic versions, this medication may serve as a cost-effective and convenient alternative to traditional systemic treatments. However, as of now, we are not aware that oral roflumilast have been submitted for approval to any regulatory agency for the indication of psoriasis; the emphasis has been on topical formulations developed by pharmaceutical companies.

##### Other Off-Label Uses of Oral Roflumilast

Roflumilast has been used off-label for several dermatological conditions (Table 4), though primarily reported as isolated case studies in the literature. Notably, there has been a growing interest in the potential use of roflumilast for Behçet’s disease (given the indication of apremilast for this condition), as well as for recurrent oral aphthosis and hidradenitis suppurativa.

An observational study including 11 patients with Behçet’s disease showed a reduction in flares and oral ulcers during treatment with roflumilast, compared to the untreated period and the previous treatment period [84]. A reduction in genital ulcers, pain and ulcer duration was also observed. AEs were reported in 54% of patients, most of which were either self-limiting or manageable through dose adjustments. No patients discontinued treatment. The same group of investigators conducted a multicenter observational study including 22 patients with severe recurrent aphthous stomatitis treated with roflumilast [86]. During roflumilast treatment, a substantial reduction in flare-ups (88%) and oral ulcers (94%) was noted compared to the untreated period. Pain and ulcer duration were also significantly reduced. AEs occurred in 13 patients, primarily headaches and gastrointestinal issues. Most side effects were either self-limiting or manageable through dose adjustments, though treatment discontinuation was required in three cases.

Ring et al. described a case of refractory hidradenitis suppurativa with axillary involvement, unresponsive to adalimumab and infliximab, that showed significant clinical improvement with roflumilast after 12 weeks, along with associated weight loss [77]. Subsequently, a retrospective study including 32 patients with hidradenitis suppurativa from a tertiary center, evaluated treatment with roflumilast 500 μg QD, showing a significant improvement in paired mean differences for IHS4-score, DLQ and VAS10 overall [83]. Eight (25%) patients required treatment discontinuation due to AEs, including nausea (n = 4), vertigo (n = 1), disease worsening (n = 2) and need for surgical intervention (n = 1).

The HERO study (NCT05682859) is a phase 4 clinical trial (n = 40) that is currently underway to evaluate the efficacy of oral roflumilast at 500 µg/day versus placebo in patients with chronic hand eczema.

Favorable outcomes and tolerance to roflumilast have also been reported for refractory nummular eczema [88], erythema annulare centrifugum [91], erythema nodosum leprosum [92], periodontitis [100] and erosive lichen planus [89,90].

#### 4.2.2. Topical Roflumilast

##### Topical Roflumilast in Psoriasis

Roflumilast cream 0.3% (ARQ-151, Zoryve^®^) has demonstrated efficacy and tolerability in treating plaque psoriasis, as shown in several clinical trials. In a phase 2b trial (NCT03638258) and two subsequent phase 3 trials (DERMIS-1 [NCT04211363] and DERMIS-2 [NCT04211389]), roflumilast 0.3% cream applied QD significantly outperformed vehicle cream [94,95]. In DERMIS-1, 42.4% of participants achieved a favorable response on the Investigator Global Assessment (IGA) scale, compared to 6.1% in the vehicle group; similar results were observed in DERMIS-2, with 37.5% improvement compared to 6.9% for the vehicle. The treatment was well tolerated, with AEs primarily mild and limited to the application site, and only a small percentage of patients discontinuing due to side effects (1% in the roflumilast group compared to 1.3% in the vehicle group).

In the open-label extension DERMIS-OLE study (NCT04286607), 24-week data showed positive outcomes for topical roflumilast in psoriasis. Of the patients who had previously used roflumilast, 84.1% completed the study, with 50% achieving “clear” or “almost clear” skin status by week 24. Safety was consistent with earlier trials; only 0.4% discontinued due to AEs, and 26.1% reported mild-to-moderate treatment-related events. Similarly, a 52-week open-label study (NCT03764475) further confirmed roflumilast’s sustained efficacy and safety in managing plaque and intertriginous psoriasis, with 45% of patients achieving an IGA rating of “clear” or “almost clear” by week 52. For participants who achieved clear skin, the average duration of response was approximately 10 months. Roflumilast was generally well tolerated; treatment-related AEs were reported in 2.7% of patients, with none classified as serious. In addition, 99% of patients showed no evidence of irritation.

The FDA granted approval for roflumilast 0.3% cream (Zoryve^®^) in July 2022 for patients aged 12 and older [11,101]. This approval was extended later in October 2023 to children aged 6 to 11, based on pharmacokinetic, efficacy and safety data from the Maximal Usage Systemic Exposure (MUSE) study (NCT04655313) [102]. Further studies are assessing its safety and effectiveness in children as young as 2 years (NCT04746911). Two recent phase 2b clinical trials have shown that 0.3% topical roflumilast is effective in managing itching and improving nighttime rest [96], as well as treating scalp areas [97].

##### Topical Roflumilast in Seborrheic Dermatitis

Recently, roflumilast foam 0.3% (ARQ-154, Zoryve^®^) has been evaluated as a therapy for seborrheic dermatitis. A phase 2a trial assessed the efficacy and safety of 0.3% roflumilast with favorable outcomes (NCT04091646) [96]. Subsequently, in a double-blinded, phase 3 trial (STRATUM, NCT04973228), 457 patients with seborrheic dermatitis were randomized to receive either roflumilast foam 0.3% or a placebo (vehicle foam) once daily for an 8-week period [98]. The primary endpoint was achieving “IGA Success” at week 8, defined as an IGA score of 0 (clear) or 1 (almost clear) with a minimum improvement of 2 points from baseline. Roflumilast proved superior to the vehicle, with 79.5% of roflumilast patients achieving the primary endpoint, compared to 58.0% in the vehicle group (*p* < 0.001). Roflumilast foam was well-tolerated, with AEs occurring in less than 3.3% of patients in both groups, most of them mild or moderate. Based on these results, in April 2023, the FDA also approved roflumilast foam 0.3% (Zoryve^®^) for the treatment of seborrheic dermatitis in adult and pediatric patients 9 years of age and older [12].

A separate 52-week study (NCT04445987) was conducted to assess the long-term safety and effectiveness of roflumilast foam 0.3% QD through 52 weeks in patients with seborrheic dermatitis. Roflumilast foam was generally well tolerated, with low rates of AEs, supporting its use for extended seborrheic dermatitis management.

##### Topical Roflumilast in Atopic Dermatitis

In July 2024, roflumilast cream 0.15% (ARQ-151; Zoryve^®^) was also approved for the treatment of AD in adults and children down to 6 years old. The approval was based on positive outcomes from a series of clinical trials, including three phase 3 trials (INTEGUMENT-1, INTEGUMENT-2 and INTEGUMENT-OLE), a phase 2 dose-ranging study (NCT03916081), and phase 1 pharmacokinetic studies.

INTEGUMENT-1 (NCT04773587) and -2 (NCT04773600) were double-blind, placebo-controlled phase 3 studies involving 1337 participants aged 6 and older with mild-to-moderate AD [99]. Both trials evaluated the application of roflumilast cream 0.15% QD. The primary endpoint was “IGA Success”, defined as an IGA score of 0 (clear) or 1 (almost clear) plus a 2-grade improvement from baseline at week 4. Significantly more patients in the roflumilast group met the primary endpoint compared to placebo (32.0% vs. 15.2% in INTEGUMENT-1; 28.9% vs. 12.0% in INTEGUMENT-2, both *p* < 0.0001). Around 40% of patients using roflumilast achieved this IGA level by week 4 in both trials, with substantial improvement as early as week 1. Significant differences were also observed in terms of itch and Eczema Area and Severity Index (EASI). By week 4, over 30% of participants on roflumilast achieved a reduction of at least 4 points in itch scores and over 40% reached a 75% reduction in EASI scores. The treatment was generally well-tolerated; AEs were mostly mild or moderate, including headache, nausea, application site pain, diarrhea, and vomiting as the most frequent ones. Over 95% of patients receiving roflumilast presented no signs of irritation at the application site.

In the INTEGUMENT-OLE open-label extension study (NCT04804605), which included 658 participants from the phase 3 trials, participants achieving clear skin after four weeks of QD application transitioned to twice-weekly use, while others continued QD. By weeks 28 and 56, 61.3% and 65.7% of participants achieved EASI-75, respectively, demonstrating sustained effectiveness over the long term.

Roflumilast cream at a lower dose of 0.05% is currently being studied in a phase 3 trial including children aged 2 to 5 years with AD (NCT04845620).

##### Off-Label Uses of Topical Roflumilast

Topical roflumilast is currently being evaluated for the treatment of papulopustular rosacea, chronic hand eczema and vitiligo in different clinical trials (Table 5).

A randomized, double-blind, placebo-controlled phase 1/2b clinical trial evaluated the safety and efficacy of roflumilast cream 0.1% and 0.3% (ARQ-252) in patients with chronic hand eczema (NCT04378569) for 12 weeks. However, results showed no statistically significant improvement in the primary endpoints for ARQ-252 over the vehicle in terms of efficacy.

A randomized, double-blind, placebo-controlled, phase 2a clinical trial (NCT04811131) was designed to evaluate the safety and efficacy of roflumilast cream 0.3% (ARQ-252) in combination with NB-UVB phototherapy in patients with non-segmental facial vitiligo. However, the study was prematurely terminated by the sponsor and the endpoints were not evaluable.

Finally, a phase 2 double-blind, placebo-controlled clinical trial is being conducted in 40 subjects with facial papulopustular rosacea and is pending result publication (NCT05278624).

### 4.3. Crisaborole (AN2728; Eucrisa^®^)

#### 4.3.1. Crisaborole in Atopic Dermatitis

Crisaborole (AN2728) is a PDE-4 inhibitor approved by the FDA and EMA for the topical treatment of mild-to-moderate AD for patients as young as 3 months of age or 2 years in the case of EMA approval (Table 6) [103,104]. Crisaborole safety and efficacy was evaluated in a phase 2, multicenter, randomized, double-blind, dose-ranging study of adolescent patients (12 to 17 years of age) affected of AD (NCT01602341) [105]. A total of 86 patients were included and randomly allocated into 4 groups to receive crisaborole 0.5% QD, crisaborole 0.5% BID, crisaborole 2% QD, and crisaborole 2% BID for 29 days. All dosing regimens led to dose-dependent improvements in Atopic Dermatitis Severity Index (ADSI) scores, as well as in all five key signs and symptoms of AD (erythema, excoriation, exudation, lichenification, and pruritus). The most significant improvements were consistently observed with crisaborole 2% topical ointment applied twice daily. With this regimen, ADSI improved by 71% from baseline, and 62% of patients achieved total or partial clearance of target lesions (ADSI ≤ 2) after 29 days of treatment. Both doses of crisaborole ointment were well tolerated, with mild application site reactions being the only treatment-related AEs [105].

Additionally, a phase 2a, randomized, double-blind, bilateral, 6-week study of crisaborole 2% topical ointment was conducted in adult patients with mild-to-moderate AD, each with two comparable target AD lesions (NCT01301508) [115]. A total of 25 patients were included and randomly assigned to apply crisaborole 2% topical ointment to one of the two target lesions and a vehicle to the other, both applied twice daily. At day 28, 17 patients (68%) demonstrated a greater reduction in ADSI scores for the active-treated lesion compared to the vehicle-treated lesion, while 5 patients (20%) showed a greater decrease in the vehicle-treated lesion than in the active-treated one. Local application-site reactions were observed in 3 patients (12%). Overall, 29 AE were reported by 11 patients, with the majority (90%) being mild and unrelated to the study medication. There were no reports of serious or severe AEs, and no patients discontinued treatment due to an AE [115].

Paller et al. reported results of two identically designed phase 3 trials in patients affected of AD aged 2 years or older (AD-301: NCT02118766; AD-302: NCT02118792) [116]. A total of 763 and 764 patients were included in both trials, respectively, and randomly allocated in a 2:1 crisaborole-vehicle manner. Compared to vehicle-treated patients, more crisaborole-treated patients achieved the Investigator’s Static Global Assessment (ISGA) score success (clear/almost clear with ≥2-grade improvement; AD-301: 32.8% vs. 25.4%, *p* = 0.038; AD-302: 31.4% vs. 18.0%, *p* < 0.001), with a greater percentage with clear/almost clear (51.7% vs. 40.6%, *p* = 0.005; 48.5% vs. 29.7%, *p* < 0.001). Treatment-related AE were uncommon and mild to moderate in severity [116].

Lastly, the CrisADe CONTROL study investigated the long-term efficacy and safety of crisaborole 2% ointment as a maintenance treatment for AD (NCT04040192) [117]. This study evaluated the effectiveness and safety of long-term, once daily crisaborole treatment compared to a vehicle. A total of 497 patients were initially treated with crisaborole 2% BID for 8 weeks. Of these, 270 patients who achieved significant improvement (clear/almost clear [IGA 0–1 with a 2-grade improvement] and ≥50% EASI improvement) were randomized to receive either crisaborole 2% QD or a vehicle during the maintenance phase. Crisaborole-treated patients showed a significantly longer median time to first flare (111 days) compared to vehicle-treated patients (30 days). Patients treated with crisaborole also experienced fewer flares (0.95 flares on average vs. 1.36) and more flare-free days (234 days vs. 199.4 days). AEs were consistent with previous clinical trials, with no serious or severe AEs reported. Therefore, once daily crisaborole may be considered a suitable long-term treatment option [117].

#### 4.3.2. Off-Label Crisaborole in Psoriasis

Crisaborole was also investigated for plaque psoriasis during its early development [102]. In a 12-week randomized, double-blind, vehicle-controlled trial (NCT01029405) using a split-body design, participants served as their own controls. Crisaborole 0.5% or 2% ointment, or a vehicle, was applied once or twice daily. The twice-daily application of Crisaborole 2% ointment demonstrated high efficacy, good tolerance, and no safety concerns. However, further development for psoriasis treatment was discontinued, and the study results were never published. Similarly, other studies supporting the use of topical crisaborole for plaque psoriasis remain unpublished [102].

More recently, a small randomized controlled trial (IRB-17-02106) with 21 patients from a single tertiary care center assessed the efficacy of crisaborole 2% ointment applied BID, compared to a vehicle, in the treatment of intertriginous, anogenital, and facial psoriasis [118]. After 4 weeks, the crisaborole group showed significant improvement in erythema, plaque elevation/induration, and scaling compared to the vehicle group. By the end of the 8-week treatment, over 70% of participants achieved lesion clearance, with no reported adverse skin reactions [118].

Despite encouraging results from small-scale studies, further investigation into the use of topical crisaborole for psoriasis has not been pursued. Even though topical crisaborole has not received regulatory approval for psoriasis treatment, its off-label use has been reported [106,107,119].

#### 4.3.3. Off-Label Crisaborole in Seborrheic Dermatitis

The efficacy and safety of crisaborole were evaluated in a phase 4 proof-of-concept clinical trial (NCT03567980), which included 30 patients with mild-to-moderate (ISGA 2 or 3) facial seborrheic dermatitis. Patients applied crisaborole 2% twice daily for 4 weeks, resulting in a significant 67.8% decrease in ISGA score by the end of treatment. Only 2% of patients experienced AEs, with only one patient discontinuing due to headache and facial pain [108].

#### 4.3.4. Other Off-Label Uses of Crisaborole

Crisaborole was recently evaluated in a phase 2a (NCT04091087), randomized, vehicle-controlled, double-blind trial for the treatment of stasis dermatitis [114]. The study included 65 participants aged 45 years or older with stasis dermatitis but no active ulceration, who received either crisaborole or vehicle (1:1) twice daily for 6 weeks. Participants treated with crisaborole showed a significantly greater reduction in total sign score from baseline compared to those receiving the vehicle, based on assessments from in-person (non-dermatologist) evaluations (−32.4% vs. −18.1%, *p* = 0.0299) and a central reader (dermatologists) assessment of photographs (−52.5% vs. −10.3%, *p* = 0.0004). AEs were primarily skin-related, with erythema, contact dermatitis, and pruritus being the most common [114].

Several case reports emphasize the broad range of inflammatory conditions that could potentially be treated with crisaborole, including lichen planus [109], porokeratosis [110], plasma cell vulvitis [111], vitiligo (NCT05298033) [113], and prurigo pigmentosa [112], among others.

Currently, clinical trials are underway to evaluate crisaborole in various dermatologic conditions (Table 7).

## 5. Oral and Topical Inhibitors of PDE4 Under Development

Oral and topical inhibitors that have been evaluated or are currently under development for dermatologic conditions are summarized in Table 8.

### 5.1. Orismilast (IBI353)

#### 5.1.1. Orismilast in Psoriasis

Orismilast is a potent oral PDE4 inhibitor with enhanced selectivity for the PDE4B and PDE4D subtypes, developed by LEO Pharma/Union Therapeutics. In a randomized, double-blind, placebo-controlled phase 2a trial involving 36 patients with moderate to severe psoriasis, participants were randomly assigned to receive either placebo or 30 mg of orismilast BID [120]. By week 16, 44.4% of patients in the orismilast group achieved PASI75, compared to 5.6% in the placebo group. The most common AEs in the orismilast group were nausea and diarrhea [120]. Subsequently, a phase 2b randomized clinical trial enrolled 202 patients to further evaluate the efficacy and safety of orismilast in moderate-to-severe plaque psoriasis (NCT05190419). Patients were randomly assigned to receive either placebo or orismilast at doses of 20 mg, 30 mg, or 40 mg BID. By week 16, all orismilast doses showed higher PASI75 achievement rates (39.5%, 49%, and 46.4%, respectively) compared to placebo (16.5%). Only three serious AEs were reported: myocardial infarction, transient ischemic attack, and erythrodermic psoriasis. Most AEs were mild or moderate, with nausea, diarrhea, and headache being the most commonly reported [120].

Currenlty, there are no ongoing clinical trials evaluating orismilast in psoriasis.

#### 5.1.2. Orismilast in Hidradenitis Suppurativa

Orismilast’s efficacy and safety were evaluated in a phase 2a, single-arm, single-center, open-label trial in patients with hidradenitis suppurativa (NCT04982432) [121]. A total of 20 patients were included and treated with 10–40 mg for 16 weeks. Only 9 patients completed the 16 weeks, as poor tolerability, mainly due to gastrointestinal AEs, led to withdrawals. As a result, an individualized approach with slower titration and a lower end dose was explored for the remaining patients. The mean AN count was reduced by 33.1% in patients who completed treatment and by 12.0% in those who discontinued. HiSCR50 was achieved by 67.0% of patients who completed treatment and 27.0% of those who discontinued. Most AEs were mild to moderate. However, further trials with more tolerable dose ranges seem warranted [121].

#### 5.1.3. Orismilast in Atopic Dermatitis

A double-blind, placebo-Controlled, phase 2b dose-ranging study will evaluate efficacy and safety of orismilast in adults with moderate to severe AD (NCT05469464). However, the trial has not started recruiting patients yet.

### 5.2. Mufemilast (Hemay005)

#### 5.2.1. Mufemilast in Psoriasis

Hemay005 is an oral non-selective PDE4 inhibitor in development developed by Tianjin Hemay Pharmaceutical Co., Tianjin, China, for the treatment of psoriasis. A randomized, placebo-controlled phase 2 trial (NCT04102241) assessed the safety and efficacy of Hemay005 in patients with moderate to severe psoriasis, enrolling 216 patients who were divided into four groups: placebo and Hemay005 at doses of 15 mg, 30 mg, and 60 mg. The trial concluded that the optimal dose of mufemilast was 60 mg BID. At 16 weeks, significant improvements were observed in PASI75 (53.6% with mufemilast vs. 16.0% with placebo, *p* < 0.0001) and sPGA success (31.3% with mufemilast vs. 6.4% with placebo, *p* < 0.0001). Most adverse events were mild and primarily gastrointestinal in nature. Additionally, a randomized, multicenter, double-blind, placebo-controlled phase 3 trial (NCT04839328) will evaluate its efficacy and safety in moderate to severe psoriasis, with patients divided into two groups: placebo and Hemay005 60 mg BID. This trial is completed, but results have not been posted yet.

#### 5.2.2. Mufemilast in Other Diseases

Mufemilast is also being evaluated for Behçet’s disease in two different trials. A phase 2 trial (NCT04609397) was terminated, and a multicenter, randomized, double-blind, placebo-controlled phase 3 trial (NCT06145893) is currently recruiting patients.

Additionally, a placebo-controlled, double-blind, randomized phase 2 trial will evaluate the efficacy and safety of mufemilast in patients with moderate to severe AD (NCT05769946). Patients were randomly assigned and treated for 12 weeks with either placebo, Hemay005 60 mg BID, or Hemay005 75 mg BID. The trial is complete, but the results have not yet been posted.

### 5.3. ME3183

ME3183 is an oral unselective PDE4 inhibitor developed by Meiji Pharma USA Inc. to treat psoriasis, with potential for use in additional inflammatory conditions like AD in the near future [131,132]. Two phase 1 studies (ME3183-1 and ME3183-2) with double-blind, placebo-controlled, single-ascending, and multiple-ascending doses assessed its safety, tolerability, and pharmacokinetics. A total of 126 healthy participants were included, and ME3183 showed safety and tolerability up to a single dose of 25 mg and up to 10 mg administered BID. The most common treatment-emergent adverse events (TEAEs) were diarrhea and headache, consistent with other approved PDE4 inhibitors, indicating no unexpected safety concerns [131,132].

In a phase 2a, multicenter, randomized, double-blind, placebo-controlled, parallel-group study involving 136 patients (NCT05268016), the safety and efficacy of ME3183 were evaluated [122]. Patients were assigned to placebo or ME3183 at doses of 5 mg BID, 7.5 mg BID, 10 mg QD, and 15 mg QD. The primary endpoint, PASI75, was assessed after 16 weeks of treatment. A significantly greater proportion of patients receiving ME3183 (5 mg BID, 7.5 mg BID, and 15 mg QD) achieved PASI75 compared to placebo (58.3%, 61.5%, and 52.0% vs. 14.8%, respectively). No difference was observed between the ME3183 10 mg QD group and placebo. Additionally, a higher percentage of patients in the ME3183 groups reached PASI90 and PASI100 than those in the placebo group. ME3183 was well tolerated, with diarrhea, nausea, and headache as the most frequent TEAEs [122].

Currently, there are no ongoing trials evaluating ME3183 in psoriasis or other inflammatory diseases.

### 5.4. Difamilast (OPA-15406)

Difamilast (OPA-15406) is a non-steroidal, selective topical PDE4 inhibitor by Otsuka Pharmaceutical Co., Tokyo, Japan, that demonstrates high selectivity for the PDE4 subtype B, although it also shows some inhibitory effects on PDE2. Despite the rapid absorption of difamilast, its plasma levels remain low on day 1 with both the 0.3% and 1% formulations [125,133]. Difamilast has only been evaluated for the treatment of AD. After positive results were observed in several phase 2 trials, it has also been evaluated in several phase 3 trials [133].

A phase 3, vehicle-controlled, randomized, double-blind, parallel-group, multicenter trial (NCT03908970) compared 1% difamilast ointment with its vehicle in Japanese patients aged 15 to 70 years who had mild-to-moderate AD [123]. The trial included a screening period of 2 to 30 days, followed by a 4-week assessment period. A total of 364 patients were randomized to receive either 1% difamilast ointment BID (n = 182) or the vehicle (n = 182) BID. At weeks 1, 2 and 4, the success rates for the IGA score (clear/almost clear with ≥2-grade improvement) were 10.99%, 23.08%, and 38.46%, respectively, in the 1% difamilast group and 0.55%, 6.04%, and 12.64% in the vehicle group (*p* < 0.001). Throughout the study, TEAEs were reported in 17.6% (32/182) of patients in the 1% difamilast group and 28.0% (12/182) in the vehicle group. Most TEAEs were mild or moderate in severity. The most common TEAEs were worsening of AD (3.8% of patients on 1% difamilast and 12.1% on the vehicle) and nasopharyngitis (4.9% on 1% difamilast and 3.8% on the vehicle). No serious AEs or deaths occurred in either treatment group [123].

An additional phase 3 (NCT03961529), long-term trial with similar characteristics included a total of 366 patients who were treated for 52 weeks [124]. Adult patients (n = 166) received difamilast 1% BID, while pediatric patients (n = 200) received either difamilast 0.3% BID (n = 144) or difamilast 1% BID (n = 56). At week 4, EASI-75 was achieved by 9.6% of adults and 32.0% of pediatric patients. These rates rose to 33.1% in adults and 60.5% in pediatric patients by week 24, and to 55.4% and 73.5%, respectively, by week 52. The cumulative success rates for the IGA score were 3.6% for adults and 15.5% for pediatric patients at week 4, 18.7% and 38.5% at week 24, and 34.9% and 52.5% at week 52. The most frequent TEAEs were worsening of AD (21.1% in adults vs. 23.5% in children) and nasopharyngitis (14.5% in adults vs. 32% in children). Severe AEs occurred in one pediatric patient (bacterial pneumonia) and in two adult patients (diffuse large B-cell lymphoma and rhegmatogenous retinal detachment); however, none were considered related to the treatment [124].

Finally, another phase 3 vehicle-controlled, randomized, double-blind, parallel-group trial (NCT03911401) included a screening period of 2 to 30 days and a 4-week assessment period [125]. A total of 251 patients were randomized in a 1:1:1 ratio to receive either 0.3% difamilast ointment (n = 83), 1% difamilast ointment (n = 85), or vehicle (n = 83), applied twice daily. By week 4, the IGA score success rates were 44.6% for 0.3% difamilast, 47.1% for 1% difamilast, and 18.1% for the vehicle (*p* < 0.001 vs. vehicle for both groups). TEAEs were mostly mild or moderate, with no serious events or deaths reported. The most common TEAEs in the treatment groups were nasopharyngitis, followed by worsening of AD and impetigo [125].

### 5.5. Lotamilast (E6005/RVT-501)

Lotamilast, also named E6005 or RVT-501, is a selective PDE4 inhibitor developed for AD by Dermavant Sciences. Dermavant Sciences developed tapinarof 1% cream for psorisasis (VTAMA^®^) and has been recently acquired by Organon [134]. In murine models of AD, topical E6005 ointment was shown to increase cyclic AMP and nerve growth factor in the skin, leading to improvement in spontaneous scratching behaviors, cutaneous nerve activity, and responses associated with itching [135,136].

A single-center study with a randomized and vehicle-controlled design investigated topical lotamilast for treating hospitalized adults with AD (NCT01179880) [126]. This study explored increasing doses of E6005 ointment, from 0.03% to 0.2%. Results indicated a positive safety profile, and the 0.2% dose demonstrated significant therapeutic effects in alleviating AD symptoms.

Subsequently, two phase 2 studies evaluating the safety and efficacy of E6005 ointment were conducted in adults and children with AD. In the former (NCT01461941), 78 adult patients were initially randomized to receive either a 0.2% E6005 ointment or a vehicle ointment for 4 weeks [127]. This was followed by an 8-week extension in which all participants received the 0.2% E6005 treatment. Over 12 weeks, E6005 showed a positive safety profile, with no serious AEs reported. E6005 levels remained undetectable in plasma, confirming its minimal systemic absorption. While initial 4-week improvements in eczema severity indices (EASI, SCORAD-objective, SCORAD-C, and pruritus scales) showed trends but weren’t statistically significant, scores after 12 weeks demonstrated significant improvements in EASI, SCORAD-objective, and SCORAD-C scores from baseline, suggesting that extended use might improve its effectiveness.

Similar trends were observed for pediatric patients aged 2–15 years old (NCT02094235) [128]. A total of 62 participants were randomized to receive either 0.05% E6005, 0.2% E6005, or a vehicle ointment BID over two weeks. The safety profile of E6005 was favorable, with no skin-related AEs reported. In terms of efficacy, the 0.2% E6005 group showed a notable but not statistically significant improvement in lesion severity and pruritus scores compared to the vehicle group. The 0.05% E6005 results, however, were similar to the vehicle.

Two additional phase 2 trials have been conducted in adults and pediatric patients, but no results have been posted (NCT02950922, NCT03394677). Currently, lotamilast remains investigational, and further studies are expected to clarify its role in both adult and pediatric populations, particularly regarding long-term effectiveness and safety.

### 5.6. LEO-29102

Leo-29102 is an orally available selective PDE inhibitor developed by Leo Pharma in 2014 [137]. Clinical development reached phase 2 trials aimed to assess efficacy and safety in AD and psoriasis treatment, respectively.

A proof of concept and dose finding phase 2 study (NCT01037881) evaluated 5 dose strengths of Leo-29102 in a 4-week, BID treatment regimen compared to a vehicle and an active comparator (Elidel cream 10 mg/g) in patients with mild-to-moderate AD. All active treatment groups achieved numerically greater improvements in the EASI scores compared to the vehicle, although without reaching statistical significance. Additionally, a dose-dependent, statistically significant reduction in both pruritus and overall disease severity was observed, based on patient assessments.

Another randomized, placebo-controlled phase 2 trial (NCT00875277) was designed to evaluate the efficacy of LEO-29102 cream and its combination with calcipotriol and betamethasone dipropionate in patients with psoriasis. The primary endpoint was the change in total clinical score of the clinical symptoms (erythema, scaling and infiltration) at week 4 compared to baseline. Results showed a significant improvement in the total clinical score in patients receiving LEO-29102 cream compared to vehicle, alone or in combination with calcipotriol or betamethasone dipropionate. No differences were seen between patients receiving betamethasone dipropionate compared to those receiving betamethasone dipropionate in combination with LEO-29102 cream, while the combination of betamethasone dipropionate or calcipotriol and LEO-29102 cream was superior to LEO-29102 cream alone. In terms of safety, the treatment was generally well tolerated, and no serious AEs were reported.

Though according to these results LEO-29102 holds potential in psoriasis and AD, no phase 3 trials are currently ongoing.

### 5.7. Pefcalcitol (M5181)

Pefcalcitol, also referred to as M5181, is a vitamin D3 analog developed by Maruho Pharmaceutical primarily for psoriasis treatment. Unlike other vitamin D3 derivatives traditionally used in dermatology, pefcalcitol demonstrates a unique mechanism by targeting PDE4 in addition to vitamin D receptors, which provides it with dual anti-inflammatory effects [138]. Preclinical studies suggested that topical pefcalcitol effectively reduces inflammation and skin lesions of plaque psoriasis while minimizing the hypercalcemic side effects often seen with other vitamin D3 analogs, providing a safer therapeutic profile for long-term use.

A phase 2 clinical trial (NCT02970331) was designed to assess the safety, tolerability, pharmacokinetics and pharmacodynamics of pefcalcitol ointment 0.005% BID 8 weeks in adolescent subjects aged 12 to 17 years old with plaque psoriasis. However, the trial was withdrawn and no patients were finally enrolled. No other clinical trials are ongoing to further evaluate topical pefcalcitol at the moment of writing.

### 5.8. PF-07038124

PF-07038124 is a potent specific PDE4B2 inhibitor developed by Pfizer and formulated for topical application using a ‘soft-drug’ approach, which allows it to be quickly inactivated upon entering the systemic circulation. The drug’s structure was based on the successful model of crisaborole. PF-07038124 possesses immunomodulatory properties demonstrated in T-cell assays, showing the capacity to inhibit IL-4 and IL-13 [129]. This mechanism suggests its potential for therapeutic use in conditions like AD and plaque psoriasis.

A randomized, double-blind, phase 2a clinical trial (EMPORIA; NCT04664153) was conducted to determine the efficacy and safety of topical PF-07038124 in adult patients with AD and plaque psoriasis [129]. The primary endpoint was the percent change from baseline in EASI and PASI, respectively. In total, 104 patients were included (70 with AD and 34 with plaque psoriasis). At week 6, results showed that PF-07038124 0.01% QD was significantly more effective than vehicle for both sets of patients. The therapy was globally well tolerated, with no reported application site reactions and comparable TEAEs across groups.

Similar results were seen in another phase 2 clinical trial (NCT05375955) including adult and adolescent patients (12 years and older) with AD and psoriasis. This randomized, double-blind study compared PF-07038124 ointment at various doses (0.01%, 0.03% and 0.06%) with the vehicle across multiple treatment arms, showing numerically greater improvement in the groups receiving higher concentrations of PF-07038124.

Additionally, phase 2a clinical trial (NCT05298033) has been recently conducted to evaluate topical crisaborole 2% and PF-07038124 0.01% alone and in combination with active narrow band UVB in vitiligo lesions, but results have not yet been posted.

### 5.9. AWD-12-281 (GW842470X)

AWD-12-281, also known as GW842470X, is a selective inhibitor of PDE4 developed by GlaxoSmithKline [139]. This compound has been primarily investigated as a dry powder inhalation in models of lung inflammation, and as a topical administration in AD and other allergic skin diseases.

At preclinical level, topical AWD-12-281 showed its anti-inflammatory effects in mouse models of cutaneous inflammation, modulating the immune response by inhibiting both Th1 and Th2 cytokine pathways [140,141].

Topical AWD-12-281 has been tested in a phase 2 clinical trial in 2006 (NCT00354510). The purpose of this randomized, double-blind, placebo-controlled study was to evaluate the efficacy and safety of 3% AWD-12-281 cream in adult patients with moderate AD. Although the study was completed, results were not posted, and no further clinical trials have been conducted or registered since then.

### 5.10. DRM02

DRM02 is a pyrazolylbenzothiazole compound developed by Dermira, that selectively inhibits several isoforms of PDE4, specifically isoforms A, B, and D [142]. In mouse models, topical application of DRM02 inhibited skin inflammation triggered by phorbol ester and reduced Th2-type hypersensitivity responses [142]. Additionally, DRM02 alleviated clinical symptoms and decreased the expression of Th17 cytokines in a psoriasis model induced by topical imiquimod. These findings suggest that DRM02 could be a promising candidate for treating inflammatory skin conditions such as psoriasis and AD [142].

Topical DRM02 has been evaluated in phase 2 trials for rosacea (NCT01993446), AD (NCT01993420), and psoriasis (NCT01993433), with no published reports.

### 5.11. HFP034

HFP034 (Chang Gung University; Kweishan, Taoyuan, Taiwan), chemically known as butyl 2-[2-(2-fluorophenyl)acetamido]benzoate, is derived from anthranilic acid compounds and demonstrates potent inhibition of the PDE4 enzyme [130]. Current studies are limited to preclinical phases, with promising efficacy and tolerability outcomes. In a mouse model of imiquimod-induced psoriasis, HFP034 applied topically reduced skin lesions and decreased epidermal thickness by limiting the expression of inflammatory cytokines and chemokines and reducing neutrophil infiltration [143]. Additional studies indicated that HFP034 raised skin cAMP levels and inhibited NF-kB activity, further supporting its anti-inflammatory effects and its potential as a therapy for psoriasis [28].

Further clinical trials are required to establish its safety and effectiveness across broader populations. However, at the moment of the writing of the present article, no clinical trials related to HFP034 are ongoing or registered.

## 6. Discussion

Treatment of moderate to severe inflammatory dermatoses often necessitates the use of immunomodulatory or immunosuppressive agents, which are frequently prescribed off-label in dermatologic practice. Although the introduction of small molecules and biologic agents has expanded therapeutic options, there remains a significant need for new immunomodulatory therapies that are both cost-effective and exhibit favorable safety profiles.

The mechanism of action of PDE4 inhibitors, based on increasing intracellular cAMP levels to reduce the release of pro-inflammatory cytokines, has proven advantageous in managing chronic inflammatory conditions. In dermatology, PDE4 inhibitors have shown particular promise in diseases such as psoriasis, AD, and seborrheic dermatitis.

Roflumilast became the first PDE4 inhibitor approved in 2010, specifically for the oral treatment of severe chronic obstructive pulmonary disease. Apremilast was the second oral PDE4 inhibitor introduced to the market, receiving initial approval in 2014 for the treatment of psoriasis, followed by approvals for psoriatic arthritis in 2014, pediatric psoriasis in 2015 and Behçet’s disease in 2019.

Apremilast, approved for moderate-to-severe psoriasis, has shown efficacy and safety across numerous clinical trials for the treatment of plaque psoriasis and other types of psoriasis, including forms affecting the palms and soles, nails, scalp, and genital area [33,34,144,145]. Apremilast is well-tolerated and approved for use in pediatric patients, making it a valuable option for various patient populations. However, its efficacy is limited and lower to that of biological agents [33,34].

Roflumilast has demonstrated efficacy as a topical treatment with a low rate of AEs and is currently approved for the treatment of psoriasis, seborrheic dermatitis, and AD. Recent studies (not sponsored by pharmaceutical companies) have shown that oral roflumilast may yield significant improvement in psoriasis. Additionally, ongoing trials are investigating its effectiveness for chronic hand eczema, rosacea, and vitiligo, suggesting a promising future for its use in various skin conditions. The pharmacokinetic and pharmacodynamic similarities between roflumilast and apremilast suggest it may be beneficial for a wider range of dermatologic diseases. Roflumilast demonstrates significantly higher potency as a PDE4 inhibitor compared to apremilast, with an IC50 value in the range of 0.2–0.7 nM versus apremilast’s IC50 of 74 nM, indicating that roflumilast achieves effective enzyme inhibition at much lower concentrations. Though roflumilast has been used off-label in different dermatologic conditions with positive outcomes, current evidence is limited, and further research is necessary to confirm its broader applications and make formal recommendations.

PDE4 inhibitors may offer several advantages over existing conventional and biologic treatments, including oral administration, no need for regular blood tests, a favorable safety and tolerability profile, and minimal contraindications. Unlike methotrexate, there are no restrictions on alcohol consumption during treatment with PDE4 inhibitors. Both apremilast and roflumilast are relatively easy to manage for both patients and healthcare providers. Most AEs of PDE4 inhibitors, such as diarrhea, nausea, and headache, are typically mild-to-moderate in severity, transient, and rarely lead to treatment discontinuation. It is essential to follow an appropriate dosing regimen, gradually increasing the dose to minimize side effects and optimize tolerability and treatment effectiveness. Both medications are relatively affordable, but roflumilast may be more economically accessible in certain regions compared to apremilast (around 30 EUR/month for the 500 µg/day oral formulation in Spain; lower than that of traditional immunosuppressants like oral cyclosporine or subcutaneous methotrexate). Among the potential advantages of roflumilast are its convenient dosing regimen and the flexibility for both topical and oral application.

Topical PDE4 inhibitors, such as crisaborole, have shown efficacy primarily in mild-to-moderate AD, with the advantage of limited systemic absorption and a relatively mild side effect profile. Crisaborole, approved for the treatment of mild-to-moderate AD for patients as young as 3 months of age, has demonstrated improvements in skin clearance, reduced itch, and favorable tolerability in both adult and pediatric populations [116,117]. However, local application-site reactions, such as burning or stinging, can be limiting factors for some patients [116,117]. Compared to topical corticosteroids and calcineurin inhibitors, crisaborole may offer a safer long-term alternative, particularly for sensitive skin areas where steroid-sparing approaches are beneficial.

All the currently approved PDE4 inhibitors (apremilast, roflumilast and crisaborole) function as a pan-PDE4 inhibitors, lacking selectivity for specific PDE4 isoforms [69,146]. There are four PDE4 subtypes (PDE4A, PDE4B, PDE4C, and PDE4D), which are expressed in around 20 different isoforms (splice variants) [18]. The specific clinical roles of different PDE4 isoforms are not yet fully understood, but PDE4B and PDE4D are thought to be key contributors to inflammatory processes, as they are the only isoforms abundantly expressed in immune cells [147,148]. Specifically, the short isoforms of PDE4B and PDE4D—particularly PDE4B2, PDE4D1, and PDE4D2—have been proposed as the crucial targets to inhibit in order to achieve anti-inflammatory effects [24]. After the approval of pan-PDE4 inhibitors, there has been renewed interest in developing next generation compounds that selectively target the PDE4B and PDE4D subtypes, such as orismilast, difamilast, PF-07038124 or DRM02. Evidence from clinical studies across multiple conditions (besides from dermatologic) suggests that PDE4B/D inhibitors demonstrate substantial efficacy, broadening the potential for these drugs in additional clinical uses.

In addition, PDE4 inhibition has shown anti-inflammatory effects in human vascular endothelial cells [149], suggesting that PDE4 inhibitors could extend benefits beyond managing chronic inflammatory skin conditions to address related comorbidities like cardiovascular disease. Roflumilast, like apremilast, may induce weight loss, which may improve metabolic profiles and reduce insulin resistance, making it an ideal option for overweight patients with skin conditions, such as those with hidradenitis suppurativa [77]. Other comorbidities that may benefit from PDE4 inhibition include inflammatory bowel disease and psoriatic arthritis, as observed with apremilast [150,151].

## 7. Conclusions

Both oral and topical PDE4 inhibitors represent valuable additions to the therapeutic armamentarium for various dermatoses, including approved indications such as psoriasis, atopic dermatitis, and seborrheic dermatitis. Emerging evidence from off-label applications highlights their potential effectiveness in managing other skin conditions. With demonstrated efficacy and a favorable safety profile, these therapies show great promise and merit further research to explore their full potential.

## 8. Future Directions

Existing evidence underscores the efficacy of PDE4 inhibitors in treating inflammatory dermatoses. Ongoing research should focus on refining their clinical use, improving patient outcomes, and minimizing adverse effects. Efforts are being made to develop new PDE4 inhibitors with enhanced specificity and tolerability, aiming to broaden their therapeutic applications and improve patient adherence. Comparative studies are needed to establish the role of PDE4 inhibitors within the treatment hierarchy, especially compared to biologics, JAK inhibitors and other small molecules, which also show significant promise. There is a need to investigate the potential for combination therapies, such as pairing PDE4 inhibitors with biologics or immunosuppressants, particularly for refractory cases where monotherapy is insufficient. Additionally, the development of more cost-effective formulations could improve accessibility and expand their use in clinical practice.

In summary, PDE4 inhibitors, both oral and topical, have emerged as versatile and effective therapeutic options for managing a wide range of dermatologic conditions. Their ability to modulate inflammatory pathways offers significant benefits in treating chronic skin diseases such as psoriasis, atopic dermatitis, and beyond. As research continues to unravel the complexities of PDE4 inhibition, these agents hold considerable promise for advancing dermatologic care and improving patient outcomes.

## Figures and Tables

**Table 1:** Uses of oral apremilast in dermatology.

Oral Apremilast		
Indicated *	Off-Label	Potential
- Adult patients with active psoriatic arthritis [31,32] - Adult patients with plaque psoriasis who are candidates for phototherapy or systemic therapy [33,34] - Pediatric patients 6 years of age and older and weighing at least 20 kg with moderate to severe plaque psoriasis who are candidates for phototherapy or systemic therapy [35] - Adult patients with oral ulcers associated with Behçet’s Disease [36]	- Atopic dermatitis [37] - Hidradenitis suppurativa [38] - Lichen planus [39] - Cutaneous lupus [40] - Morphea [41] - Sarcoidosis [42] - Anti-laminin γ1 (p200) pemphigoid [43] - Central centrifugal cicatricial alopecia (NCT03521687) - Vulvodynia (NCT00814632) - Dermatomyositis (NCT03529955) - Erythema nodosum leprosum [44] - Pemphigus foliaceous and vulgaris [45,46] - Hailey-Hailey [47] - Frontal fibrosing alopecia (NCT03422640)	- Palmoplantar pustulosis (NCT05174065) - Vitiligo (NCT03036995; NCT06593197) - Erosive/oral lichen planus (NCT03656666, NCT06260904) - Acne conglobata (NCT04161456) - Erythema multiforme (NCT05875714) - Epidermolysis bullosa simplex generalized (NCT06509984)

* Indicated both by the Food and Drug Administration (FDA) and the European Medicines Agency (EMA).

**Table 2:** Most relevant real-world studies of apremilast.

Study	n	Type of Patients	Type of Study	Results	Most Frequent Adverse Events
D. Ioannides et al. [56]	287	Bio-naïve patients with moderate psoriasis	Observational prospective study	At 52 weeks: - PASI75 61% - PASI90 32.1%	Loss of weight, diarrhea and headache
E. del Alcázar et al. [55]	377	Moderate to severe plaque psoriasis and palmoplantar psoriasis	Retrospective observational study	At 52 weeks: - PASI75 49.7% - PASI90 26.5% - PPPGA score of 0/1 83%	Diarrhea, gastrointestinal symptoms, nausea, headache and asthenia
T. Graier et al. [57]	367	Moderate to severe psoriasis	Observational retrospective study	At 52 weeks: - PASI75 56.4% - PASI90 38.2%	Gastrointestinal symptoms, headache and infection
G. Licata et al. [58]	231	Moderate to severe plaque psoriasis	Retrospective observational study	At 52 weeks: - PASI75 97% - PASI90 89.3% - PASI100 62%	Gastrointestinal symptoms, weight loss, and headache

Abbreviations: PASI, Psoriasis Area and Severity Index; PPPGA, Palmo-Plantar Psoriasis Global Assessment.

**Table 3:** Ongoing clinical trials with oral apremilast in dermatology.

NCT	Indication	Intervention	Clinical Stage	Primary Endpoint
NCT05863273	Psoriasis	Apremilast 30 mg BID	Observational	Proportion of patients with DLQI improvement (DLQI ≤ 5 or DLQI improvement by ≥4 points from baseline) or PASI improvement (PASI < 3) at 16 weeks of treatment
NCT05601492	Psoriasis: mild	Apremilast	Early phase 1	Adherence for 6 months
NCT05565560	Psoriasis: Pediatric patients with moderate to severe psoriasis	Apremilast 20 mg BID (weight < 50 kg) or apremilast 30 mg BID	Phase 3	Achievement of a static sPGA of clear (0) or almost clear (1) with at least 2 points reduction from baseline and PASI75 at Week 16
NCT06088199	Psoriasis: Pediatric patients with moderate to severe psoriasis	Apremilast dosed by participants body’s weight	Phase 3	Number of Participants with TEAE
NCT05744466	Psoriasis: Comparison between apremilast vs. deucravacitinib in real-world settings	Observation of patients who have initiated deucravacitinib and patients who have initiated apremilast	Observational	Change in BSA at 60 months
NCT05174065	Palmoplantar pustulosis	Apremilast 30 mg BID compared to placebo	Phase 3	Number of participants achieving at least 50% reduction from baseline in PPPASI total score (PPPASI-50) at week 16
NCT04528082	Oral ulcers in Behçet’s disease in pediatric patients	Apremilast or placebo for 12 weeks, followed for 40 weeks of apremilast treatment	Phase 3	Area Under the Curve for the Number of Oral Ulcers from Week 0 Through Week 12
NCT06260904	Oral lichen planus	Apremilast 30 mg BID compared to methotrexate	Phase 4	Pain by VAS score at 8 and 12 weeks
NCT03656666	Female genital erosive lichen planus	Apremilast 30 mg BID vs. placebo for 24 weeks	Phase 2	Mean GELP score at week 24 in apremilast-treated patients versus placebo-treated patients
NCT06593197	Vitiligo	Apremilast 30 mg BID with NBUVB compared to NBUVB for 8 months	Phase 4	Determine by VASI score the frequency of patients halted their progression and extent of repigmentation at 8, 16, 24 and 32 weeks
NCT04161456	Acne conglobata	Apremilast 30 mg BID	Phase 2	Proportion of subjects who achieve at least a 50% reduction in total number of inflammatory lesions after 24 weeks of treatment
NCT05875714	Erythema multiforme (EM)	Apremilast 30 mg BID	Phase 2	Change in frequency and duration of EM flares at 24-week evaluation.
NCT06509984	Epidermolysis bullosa simplex generalized	Apremilast	Phase 2	Number of new blisters counting every day for 20 weeks

Abbreviations: DLQI, Dermatology Life Quality Index; PASI, Psoriasis Area and Severity Index; sPGA, Physician Global Assessment; TEAE, Treatment Emergent Adverse Events; BSA, Body Surface Area; PPPASI, Palmoplantar Pustulosis Area and Severity Index; VAS, Visual Analogue Scale; GELP, Genital Erosive Lichen Planus; VASI, Vitiligo Area Scoring Index.

**Table 4:** Uses of oral and topical roflumilast in dermatology.

***Oral Roflumilast***		
**Indicated**	**Off-Label**	**Potential**
None	- Psoriasis [73,74,75,76,78] - Hidradenitis suppurativa [77,83] - Behçet disease [84,85] - Recurrent oral aphtosis [86,87] - Nummular eczema [88] - Erosive lichen planus [89,90] - EAC [91] - Erythema nodosum leprosum [92]	- Chronic hand eczema (NCT05682859) - Epidermolysis Bullosa Acquisita [93]
***Topical Roflumilast***		
**Indicated**	**Off-Label**	**Potential**
- Zoryve^®^ cream (0.3%) is FDA-approved for plaque and inverse psoriasis, for patients aged 6 and older [94,95,96,97] - Zoryve^®^ cream (0.3%) is FDA-approved for seborrheic dermatitis in adult and pediatric patients 9 years of age and older [98] - Zoryve^®^ cream (0.15%) is FDA-approved for mild to moderate atopic dermatitis in adult and pediatric patients 6 years of age and older [99]	None published	- Chronic hand eczema (NCT04378569) - Vitiligo (NCT04811131) - Rosacea (NCT05278624)

Abbreviations: FDA, Food and Drug Administration.

**Table 5:** Ongoing clinical trials with oral and topical roflumilast in dermatology.

***Oral Roflumilast***					
**Dermatosis**	**NCT**	**Phase**	**n**	**Primary Endpoint**	**Results**
Psoriasis	NCT04549870 (PSORRO trial) [79]	2	46	Efficacy (PASI75 at week 12) and safety of oral roflumilast vs. placebo for plaque psoriasis	At week 12, PASI75 was achieved by 35% of roflumilast patients and 0% of placebo patients (*p* = 0.014). Common AEs included gastrointestinal symptoms, weight loss, headaches, and insomnia. Three patients required therapy discontinuation (roflumilast n = 2; placebo, n = 1).
	NCT05684744 [82]	2–3	30	Efficacy (PASI75 and SmPASI at week 12) and safety of oral roflumilast compared to intramuscular MTX for plaque and scalp psoriasis	After 12 weeks, both groups demonstrated a statistically significant reduction in all assessed scores. Greater PASI and SmPASI responses were observed in the roflumilast group, although without reaching statistical significance. Common AEs included gastrointestinal symptoms in both groups; also fatigue and anemia in the MTX group. No therapy discontinuation was required.
Chronic hand eczema	NCT05682859	4	40	Efficacy (HECSI75 at week 16) and safety of oral roflumilast vs. placebo for chronic hand eczema	Recruiting—no results posted
***Topical Roflumilast***					
**Dermatosis**	**NCT**	**Phase**	**n**	**Primary Endpoint**	**Results**
Atopic dermatitis in children aged 2 to 5 years	NCT04845620	3	652	IGA success, defined as a IGA score of ‘clear’ or ‘almost clear’ plus a 2-grade improvement from baseline at week 4.	Completed—Results to be posted
Chronic hand eczema	NCT04378569	1–2	230	- IGA score of ‘clear’ or ‘almost Clear’ at week 12 - Number of participants with ≥1 AE at week 3 - Number of participants with an application site reaction at week 3	- At week 12, no statistically significant improvement in IGA score was achieved in the ARQ-252 cream groups over the vehicle group. - At week 3, AEs were reported in 1 (14.3%) of 7 patients in the ARQ-252 cream 0.3% QD group. - No application site reactions were reported in any of the 7 patients.
Rosacea	NCT05278624	2	40	Reduction in rosacea facial inflammatory lesion count from baseline as compared to week 12.	Completed—Results to be posted

Abbreviations: AE, adverse events; PASI75, 75% reduction in Psoriasis Area and Severity Index compared to baseline; SmPASI, scalp-modified PASI; MTX, methotrexate, HECSI75, 75% reduction in Hand Eczema Severity Index compared to baseline; IGA, Investigator’s Global Assessment.

**Table 6:** Uses of topical crisaborole in dermatology.

*Topical Crisaborole*		
Indicated	Off-Label	Potential
- Eucrisa^TM^ ointment (2%) is FDA-indicated for topical treatment of mild to moderate atopic dermatitis in adult and pediatric patients 3 months of age and older [103] - Staquis^®^ ointment (2%) is EMA-indicated for the treatment of mild to moderate atopic dermatitis in adults and paediatric patients from 2 years of age with ≤40% body surface area affected [104]	- Psoriasis [106,107] - Seborrheic dermatitis [108] - Lichen planus [109] - Porokeratosis [110] - Plasma cell vulvitis [111] - Prurigo pigmentosa [112]	- Skin toxicity induced by cetuximab (NCT06118047) - Vitiligo (NCT05298033) [113] - Stasis dermatitis (NCT04091087) [114]

Abbreviations: FDA, Food and Drug Administration; EMA, European Medicines Agency.

**Table 7:** Ongoing clinical trials with topical crisasaborole in dermatology.

NCT	Indication	Intervention	Phase	Primary Endpoint
NCT06118047	Skin toxicity induced by cetuximab	Crisaborole 2% ointment in patients with acneiform eruption due to cetuximab	2	Remission rate of cetuximab-related acneiform eruption at 8 weeks
NCT05298033	Vitiligo	Crisaborole 2% ointment or PF-07038124 with or without NBUVB	2	Proportion of participants achieving at 50% or greater improvement from baseline in F-VASI
NCT04800185	Atopic dermatitis: Skin microbiome change	Crisaborole 2%	1	Determine a change in skin microbiome taxonomic units after 16 weeks of treatment with crisaborole in atopic dermatitis

Abbreviations: NBUVB, Narrowband ultraviolet B; F-VASI, Facial Vitiligo Area Scoring Index.

**Table 8:** Overview of PDE4 inhibitors approved and under clinical development in dermatology.

PDE4 Inhibitor	Chemical Structure	PDE4 Prolife	IC_50_	Sponsor	Formulation	Condition	Phase of Development	NCT Number
**Apremilast**	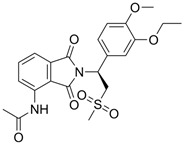	Unselective PDE4 inhibitor	74 nM	Amgen, Inc. (Thousand Oaks, CA, USA)	Oral	Psoriasis	Approved by FDA and EMA	
						Psoriatic arthritis	Approved by FDA and EMA	
						Behçet’s disease	Approved by FDA and EMA	
						Others	See Table 3	
**Roflumilast**	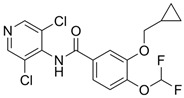	Unselective PDE4 inhibitor	0.2–0.7 Nm *	AstraZeneca (Cambridge, UK)	Oral and Topical	Psoriasis	Approved by FDA (topical)	
						Seborrheic dermatitis	Approved by FDA (topical)	
						AD	Approved by FDA (topical)	
						Others	See Table 5	
**Crisaborole**	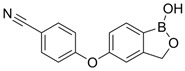	Unselective PDE4 inhibitor	490 nM	Pfizer, Inc. (New York City, NY, USA)	Topical	AD	Approved by FDA and EMA	
						Others	See Table 7	
**Orismilast (LEO-32731)**	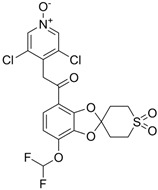	Selective PDE4B/D inhibitor	3–104 * nM	LEO Pharma; Union Therapeutics (Hellerup, Denmark)	Oral	Psoriasis	2a/b	NCT05190419; NCT02888236 [120]
						AD	2b	NCT05469464
						Hidradenitis suppurativa	2a	NCT04982432 [121]
**Mufemilast** **(Hemay005)**	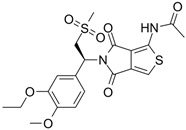	Unselective PDE4 inhibitor	80–120 nM	Tianjin Hemay Pharmaceutical Co., Ltd. (Tianjin, China)	Oral	Psoriasis	3	NCT04839328
						AD	2	NCT05769946
						Behçet’s disease	3	NCT06145893
**ME3183**	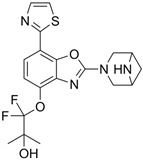	Unselective PDE4 inhibitor	1.28–2.33 nM	Meiji Pharma USA Inc. (Santa Ana, CA, USA)	Oral	Psoriasis	2a	NCT05268016 [122]
**Difamilast (OPA-15406)**	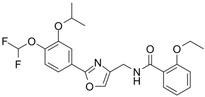	Selective PDE4B/PDE2 inhibitor	11.2 nM	Otsuka Pharmaceutical Co., Ltd. (Osaka, Japan)	Topical	AD	3	NCT03908970 [123]; NCT03961529 [124]; NCT03911401 [125]
**Lotamilast (E6005/RVT-501)**	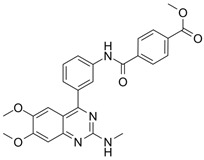	Unselective PDE4 inhibitor	2.8 nM	Dermavant Sciences; Eisai Co., Ltd. (Phoenix, AZ, USA)	Topical	AD	2	NCT01179880 [126]; NCT01461941 [127]; NCT02094235 [128]; NCT02950922; NCT03394677
**LEO-29102**	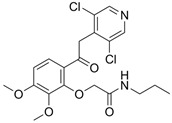	Unselective PDE4 inhibitor	5 nM	Leo Pharma (Ballerup, Denmark)	Topical	AD	2	NCT01037881
						Psoriasis	2	NCT00875277
**Pefcalcitol (M5181)**	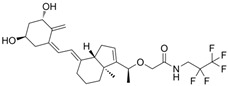	Vitamin D3 analog and unselective PDE4 inhibitor	Not reported	Maruho Pharmaceutical (Osaka, Japan)	Topical	Psoriasis	2	NCT02970331
**PF-07038124**	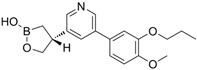	Selective PDE4B2 inhibitor	0.5 nM	Pfizer Inc. (New York, NY, USA)	Topical	Psoriasis and AD	2	NCT04664153 [129]; NCT05375955
						Vitiligo	2	NCT05298033
**AWD-12-281 (GW842470X)**	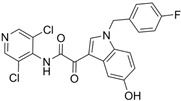	Unselective PDE4 inhibitor	9.7 nM	GlaxoSmithKline (Brentford, UK)	Topical	AD	2	NCT00354510
**DRM02**	Undisclosed	Selective PDE4A/B/D inhibitor	0.6–14 µM	Dermira (Menlo Park, CA, USA)	Topical	Psoriasis	2	NCT01993433
						AD	2	NCT01993420
						Rosacea	2	NCT01993446
**HFP034**	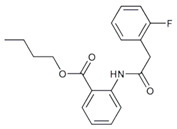	Unselective PDE4 inhibitor	4.2 μM	Chang Gung University (Kweishan, Taoyuan, Taiwan)	Topical	Psoriasis	Preclinical	[130]

Abbreviations: PDE4, Phosphodiesterase-4; IC_50_, half maximal inhibitory concentration (defined as the concentration of the drug required to inhibit a specific biological function by 50%); AD, atopic dermatitis. * Depending on the specific PDE4 isoform targeted.
